# Mechanism modeling and application of *Salvia miltiorrhiza* percolation process

**DOI:** 10.1038/s41598-023-35529-2

**Published:** 2023-05-23

**Authors:** Wanying Wang, Feng Ding, Haibin Qu, Xingchu Gong

**Affiliations:** 1 Pharmaceutical Informatics Institute, College of Pharmaceutical Sciences, Zhejiang University, Hangzhou, 310058 China; 2 Innovation Center in Zhejiang University, State Key Laboratory of Component-Based Chinese Medicine, Zhejiang University, Hangzhou, 310058 China; 3 Jinhua Institute of Zhejiang University, Jinhua, 321016 China

**Keywords:** Chemical engineering, Chemical engineering

## Abstract

Percolation is a common extraction method of food processing industry. In this work, taking the percolation extraction of salvianolic acid B from *Salvia miltiorrhiza *(*Salviae Miltiorrhizae Radix et Rhizoma*) as an example, the percolation mechanism model was derived. The volume partition coefficient was calculated according to the impregnation. experiment. The bed layer voidage was measured by single-factor percolation experiment and the internal mass transfer coefficient was calculated by the parameters obtained by fitting the impregnation kinetic model. After screening, the Wilson and Geankoplis, and Koch and Brady formulas were used to calculate the external mass transfer coefficient and the axial diffusion coefficient, respectively. After substituting each parameter into the model, the process of percolation of *Salvia miltiorrhiza* was predicted, and the coefficient of determination R^2^ was all greater than 0.94. Sensitivity analysis was used to show that all the parameters studied had a significant impact on the prediction effect. Based on the model, the design space including the range of raw material properties and process parameters was established and successfully verified. At the same time, the model was applied to the quantitative extraction and endpoint prediction of the percolation process.

## Introduction

Percolation is a common extraction method of traditional Chinese medicine. Generally, the extract is placed in a percolator, the solvent is continuously added in the percolator, and the solution after extraction is collected at the same time. Among the 1609 preparations contained in the 2020 edition of the Chinese Pharmacopoeia (Part I), about 9.8% of the preparations involved the percolation process, with a total of 157 species^1^. The advantages of percolation are that the equipment is simple, and the operation is easy. The advantage of percolation for extracting traditional Chinese medicine is that the process is mild, which is conducive to obtaining thermally unstable components or components, etc. The disadvantage is that the solvent consumption is large and the time of extraction is long^2^.

The current modeling methods for the percolation process include statistical modeling and mechanistic modeling. Statistical modeling is the most commonly used modeling method in percolation process research.

Cao Hui used the Box–Behnken design to study the effects of ethanol concentration, immersion time, and percolation flow rate on the total saponin content and total saponin extraction rate of the percolation process. The second-order polynomial modeling was used, and the model was further used to optimize the process parameters^3^. Chen Weilin et al. investigated the three parameters of ethanol concentration, solvent dosage, and percolation flow rate in the percolation extraction process of Qixue Shuangbu Tinctura with a Box-Behnken design, and established a second-order polynomial model with the concentration of the index components and extracts as the inspection indicators, then optimized the parameters^4^. The modeling method of the above work is simple, and the calculation is easy, but the influence of the difference in the quality of medicines between batches is not considered.

Wang Xiaoyu adopted the idea of feedforward control to optimize the process parameters of Sophora flavescens Alt. and Heterosmilax japonica Kunth percolation. They used a second-order polynomial to establish a quantitative model among the quality information of raw material, process parameters and percolation process effect. Then the quality information of new batches of medicinal pieces was put into the model, so that the optimized percolation process parameters can be calculated^5^. The idea is to adjust the process parameters according to the fluctuation of the quality of the raw material between batches, which is conducive to stabilizing the quality of the percolation extract between batches. This method can reflect the quality difference of raw material in the model, but it is difficult to apply to different percolation equipment.

Strube's research group from Clausthal University of Technology has conducted in-depth research on the percolation process mechanism modeling. The percolation process mechanism model has been established to simulate the percolation extraction process of pepper berries and vanilla beans, and good prediction results have been achieved^6^. They used scanning electron microscopy, Raman/infrared imaging and other technologies to study and determine the parameters such as the porosity and particle size distribution of the medicinal materials. And they measured the content of the target components in the medicinal materials and the impregnation equilibrium curve and used tracer experiments to determine the axial diffusion coefficient and bed layer voidage. Finally the above parameters were used for model calculations^7^. They compared the effect of using Fick's law and Maxwell–Stefan equation to simulate the effect of diffusion in the water extraction process, and found that the data predicted by the Maxwell–Stefan equation has higher accuracy^8^. The research group has conducted in-depth research on the mechanism modeling of the percolation process. The established mechanism model can reflect the essence of the percolation process and has the advantages of being applicable to different batches of medicinal materials and different equipment, which is conducive to improving the understanding of the production process. However, the measurement methods of the parameters such as particle size and bed layer voidage of the medicinal materials are relatively complicated, and the requirements for instruments and equipment are relatively high.

*Salvia miltiorrhiza (Salviae Miltiorrhizae Radix et Rhizoma)* is a commonly used Chinese materia medica for promoting blood circulation and removing blood stasis. It has a wide range of pharmacological effects and is mainly used for the treatment of irregular menstruation, palpitation, insomnia and various cardiovascular diseases, particularly angina pectoris and myocardial infarction^9^. Its water-soluble active ingredient salvianolic acid B (SAB) is easily degraded at high temperature^10^. Therefore, it is more suitable to extract SAB by percolation method. In this work, taking the percolation and extraction of *Salvia miltiorrhiza* as an example, the process mechanism modeling research of percolation process was carried out. The method of parameter measurement and calculation was discussed, and the design space of the parameters was obtained by calculation using the established mechanism model, and the quantitative extraction and endpoint control methods were proposed, which were verified with new batches of medicinal materials.

## Materials and methods

### Materials and chemicals

Sources of different *Salvia miltiorrhiza* was shown in Table 1, SAB reference substance (Lot number: 200602, purity ≥ 98%) was supplied by Shanghai Winherb Pharmaceutical Technology Development. HPLC-grade acetonitrile and HPLC-grade methanol were obtained from Merck (Darmstadt, Germany). No specific permissions were required for the described field studies. The collection of plant material and the performance of experimental research on such plants complied with the national guidelines of China. Batches D1 to D4 were used for model building and fitting, and batches D5 were used for model validation.

**Table 1:** Sources of different *Salvia miltiorrhiza.*

Batch of *Salvia miltiorrhiza*	Manufacturer	Place of origin
D1	Bozhou Yonggang Medicinal Pieces Factory Co., Ltd.	Shanxi
D2	Hebei Kangan Biological Technology Co., Ltd.	Shandong
D3	Tonghua Tongfu Ginseng Products Co., Ltd.	Sichuan
D4	Bozhou Yonggang Medicinal Pieces Factory Co., Ltd.	Shanxi
D5	Anhui Hong Entang Biotechnology Co., Ltd.	Yunnan

### Determination of impregnation kinetic curve

*Salvia miltiorrhiza* is crushed with a medicinal material grinder (DFY-200, Wenling Linda Machinery Co., Ltd.). 15.0 g of *Salvia miltiorrhiza* powder was put into a conical flask, and 90.0 g of Ultra-pure water (Milli-Q, Millipore) was added. The batch of medicinal herbs and the particle size of the Chinese medicine tablets were used as variables and multiple groups of experiments were carried out in parallel. The impregnation was terminated at different time points respectively. Filtration was carried out to obtain *Salvia miltiorrhiza* extract. The SAB in the extract of *Salvia miltiorrhiza* was detected by high performance liquid chromatography (HPLC) (1100 System, Agilent Technologies). The measured impregnation kinetic curves were fitted by Formulas (1)–(3) respectively.

First order kinetic model^11^:

1 $$C={C}_{eq}\left(1-{e}^{-kt}\right)$$

where, *C* represents concentration, ${C}_{eq}$ represents Concentration of SAB in solution at immersion equilibrium, *k* represents overall extraction rate constant, $t$ represents time.

Peleg’s model^12^:

2 $$C=\frac{t}{\left({k}_{1}+{k}_{2}t\right)}$$

where, *k*_*1*_ represents Peleg’s rate constant, *k*_*2*_ represents Peleg’s capacity constant.

Diffusion model^13^:

3 $$C={C}_{eq} \left[1-\frac{6}{{\pi }^{2}}\sum_{n=1}^{\infty }\frac{1}{{n}^{2}}\mathrm{exp}\left(-\frac{{D}_{eff}{n}^{2}{\pi }^{2}t}{{r}^{2}}\right) \right]$$

where, *D*_*eff*_ represents apparent diffusion coefficient, $r$ represents medicinal particle radius. In this work, only the first three items of the infinite summation are taken.

### Extraction and determination of SAB in* Salvia miltiorrhiza*

The steps of ultrasonic extraction method are as follows^1^. 0.15 g of *Salvia miltiorrhiza* powder (passed through a No. 3 sieve) was accurately weighed and put in a conical flask with cover. 50 mL of methanol–water (8:2,v/v) mixed solution was added accurately and weighed. After ultrasonicated (50W, 40 kHz) for 30 min and cooled, the methanol–water (8:2, v/v) mixed solution was weighed again and the volume of solution was adjusted to 50 mL. After filtered, the SAB concentration in the filtrate was determined by HPLC.

The steps of multiple impregnation method are as follows. 20.0 g of a certain particle size *Salvia miltiorrhiza* powder was put into a conical flask, with about 20 times of water was added. This conical flask was placed in a water bath thermostatic oscillator (ZBR-CD-19, Shanghai Lichen Bangxi Instrument Technology Co., Ltd.) at 25 °C for 2 h. After filtration, the filtrate was collected. 10 times of water to the medicinal materials was continued to be added into conical flask for impregnation for 2 h. This step repeated 3 times. Finally, The SAB content in the last impregnation solution was determined by HPLC. All the filtrates were integrated together. Then the filtrate volume was determined, and the SAB content in the filtrate was also determined by HPLC.

### Determination of volume partition coefficient of medicinal materials

*Salvia miltiorrhiza* powder and water with different solid-to-liquid ratios were added to conical flasks, placed in a water bath thermostatic oscillator at 25 °C for 18 h. Then the mixture was filtered with gauze to separate the extract from the medicinal materials, and the quality of the medicinal materials and the quality and volume of the extract were measured respectively. The content of SAB in the extract was determined by HPLC, and the volume partition coefficient of medicinal materials was calculated by Formulas (4)–(6).

According to the law of conservation of mass, we can get the result:

4 $${C}_{w}{V}_{w}+{C}_{s}{V}_{s}={M}_{0}m$$

where, *V* is volume, $C$ is concentration, subscripts *w* and *s* refer to solutions and medicinal materials respectively; *M*_*0*_ is SAB content of *Salvia miltiorrhiza*, and *m* is medicinal mass.

It is assumed that the interior of the medicinal material particles is uniform, and the SAB concentration inside the medicinal material granules and the outer surface of the medicinal material is linearly related. When the kinetics equilibrium is reached, the concentration of the surface solution of the medicinal material was considered to be equal to the concentration of the whole solution. Therefore, the Formula for calculating the volume partition coefficient (${D}_{is}$) was as follows:

5 $${D}_{is}=\frac{{C}_{s}}{{C}_{w}}$$

Therefore, the final calculation Formula of ${D}_{is}$ was shown in Eq. (6):

6 $${D}_{is}= \left(\frac{{M}_{0}m}{{C}_{w}}-{V}_{w} \right)/{V}_{s}$$

### Percolation experiment of *Salvia miltiorrhiza*

*Salvia miltiorrhiza* was crushed, then passed through different size meshes. A certain mesh range (the particle size range of *Salvia miltiorrhiza* powder was expressed in the range of mesh size below) of the *Salvia miltiorrhiza* powder was impregnated with about 6 times the amount of water. After fully swollen, it was put into a percolation cylinder with a diameter of 5 cm and immersed for 12 h. Afterwards, water was used as solvent, and percolation was carried out at a certain flow rate (peristaltic pump, BL100, Changzhou Vesil Fluid Technology Co., Ltd.). The percolation solution at different times was collected and determined by HPLC to obtain the percolation process curve. According to the experimental conditions in Table 2, the single-factor percolation experiment of *Salvia miltiorrhiza* was carried out.

**Table 2:** Experimental conditions of single factor percolation extraction.

Experiment	Batch of *Salvia miltiorrhiza*	Particle size (mesh number of sieve)	Flow rate (mL/min)	Weight of medicinal material(g)
E1	D4	10–24	2.0	50.0
E2	D4	10–24	2.0	30.0
E3	D4	10–24	2.0	70.0
E4	D4	10–24	3.0	50.0
E5	D4	10–24	4.0	50.0
E6	D4	5–10	2.0	50.0
E7	D4	24–50	2.0	50.0
E8	D1	10–24	2.0	50.0
E9	D3	10–24	2.0	50.0

### Analysis method

#### Analysis of SAB in *Salvia miltiorrhiza* by HPLC^14^

**Figure 1:**
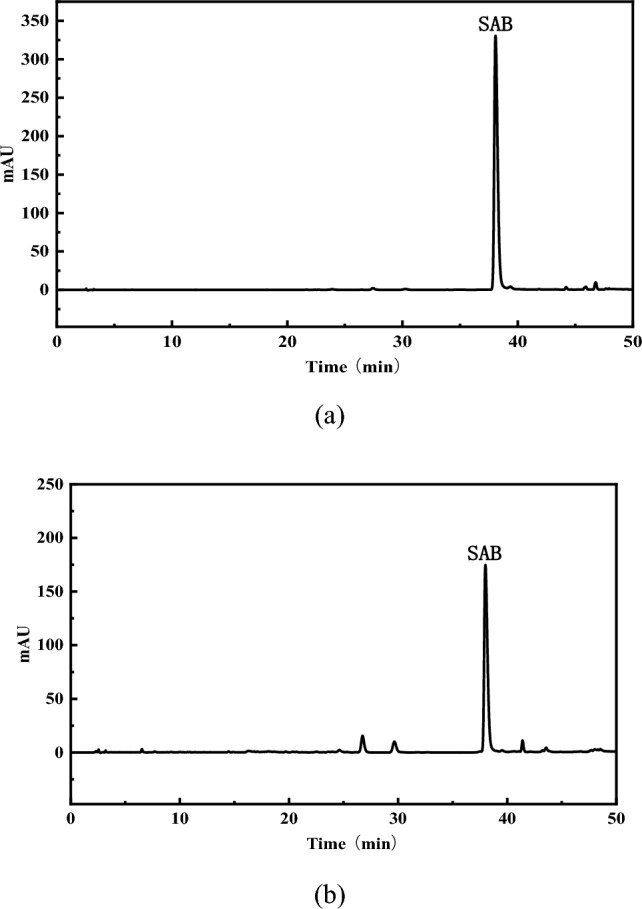
Liquid chromatography of SAB. (**a**) Chromatogram of SAB reference substance; (**b**) chromatogram of *Salvia miltiorrhiza* percolation solution sample.

Analyses were performed on Agilent Extend-C18 column (5 μm, 4.6 × 250 mm) with the column temperature controlled at 25 °C; The solvent flow rate was maintained at 1 mL/min, while the sample injection volume was set at 5 μL. The detection wavelength was 281 nm. The mobile phase was consisted of solvent A (0.1% (v/v) formic acid in water) and solvent B (acetonitrile). The solvent gradients were as follows: 0–10 min 7–17% B, 10–16 min 17–21% B, 16–32 min 21–21% B, 32–40 min 21–29% B, 40–44 min 29–35% B, 44–50 min 35–72% B, 50–55 min 72–30% B. The typical chromatogram obtained was shown in Fig. 1.

### Mechanism model of percolation process

**Figure 2:**
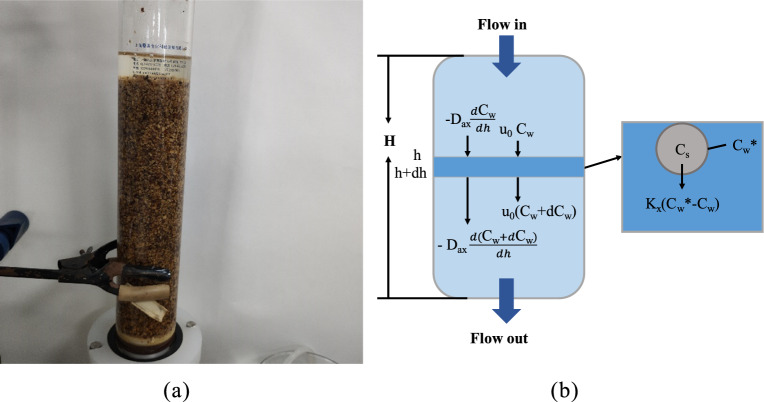
Schematic diagram of mass transfer in a percolation column. (**a)** Diagram of percolation device; (**b**) Schematic diagram of percolation. (*h* is the height of the percolation column, *u*_*0*_ is the flow rate, *D*_*ax*_ is axial diffusivity, *K*_*x*_ is the mass transfer coefficient, ${C}_{w}^{*}$ is concentration of ingredients on the outer surface of medicinal material particles).

The derivation process refers to the mass transfer process in column chromatography^15^. Take an infinitesimal element of microelements from the percolation column for analysis, as shown in Fig. 2.

As shown in Fig. 2b, for an infinitesimal element in the percolation column to perform constant material calculation, the change of the SAB concentration was affected by three factors: solvent flow, axial diffusion and mass transfer. According to the principle of mass conservation, Formula (7) can be obtained.

7 $$\frac{d{C}_{w}}{dt}=-{u}_{0}\frac{d{C}_{w}}{dh}+{D}_{ax}\frac{{d}^{2}{C}_{w}}{d{h}^{2}}+\frac{\left(1-\varepsilon \right)}{\varepsilon }a{K}_{x}\left({C}_{w}^{*}-{C}_{w}\right)$$

where, *ε* is the bed porosity, $a$ is the specific surface area of medicinal particles. Assuming that the medicinal material particles are ideal spherical, the formula for calculating the specific surface area is:$a=\frac{3}{r}$. Therefore, Formula (7) can be transformed into Formula (8).

8 $$\frac{d{C}_{w}}{dt}=-{u}_{0}\frac{d{C}_{w}}{dh}+{D}_{ax}\frac{{d}^{2}{C}_{w}}{d{h}^{2}}+\frac{3}{r}\frac{\left(1-\varepsilon \right)}{\varepsilon }{K}_{x}\left({C}_{w}^{*}-{C}_{w}\right)$$

The initial condition of the above Formula is: the concentration of the aqueous phase is constant before entering the percolation column. We can get Formula (9).

9 $${C}_{w}\left(z=0,t\right)={C}_{w}^{inlet}$$

The boundary conditions are: the concentration of the water phase will not change after it leaves the seepage column. We can get Formula (10).

10 $$\frac{\partial {C}_{w}(z=H,t)}{\partial t}=0$$

Similarly, for the analysis of the medicinal material phase in an infinitesimal element of the percolation column, the concentration of SAB in the medicinal material granules changes to the concentration that enters the water phase through interphase mass transfer. We can get Formula (11).

11 $$\frac{d{C}_{s}}{dt}=\frac{3}{r}{K}_{x}({C}_{w}-{C}_{w}^{*})$$

Assuming that the interior of the medicinal material particles is uniform, the solid–liquid equilibrium relationship is a simple linear relationship. Therefore, we can get Formula (12).

12 $${C}_{w}^{*}=\frac{{C}_{s}}{D}$$

From above all, we can get Formula (13).

13 $$\frac{d{C}_{s}}{dt}=\frac{3}{r}{K}_{x}({C}_{w}-\frac{{C}_{s}}{{D}_{is}})$$

According to the above formulas, Matlab software is used to build a percolation model to predict the percolation process. In order to evaluate the prediction effect of the model. The coefficient of determination R^2^ was chosen to evaluate the model. The formula for the coefficient of determination R^2^ is as follows:

14 $${\mathrm{R}}^{2}=1-\frac{\sum_{i=1}^{j}{\left({y}_{i}-\widehat{{y}_{i}}\right)}^{2}}{\sum_{i=1}^{n}{\left({y}_{i}-\overline{{y }_{i}}\right)}^{2}}$$

where, ${y}_{i}$ is the measured value of i point in the percolation curve, $\overline{{y }_{i}}$ is the average value of the measured values of all points on the percolation curve, $\widehat{{y}_{i}}$ is the predicted value of the i point in the percolation curve, j is the number of points.

### Calculation of design space

**Figure 3:**
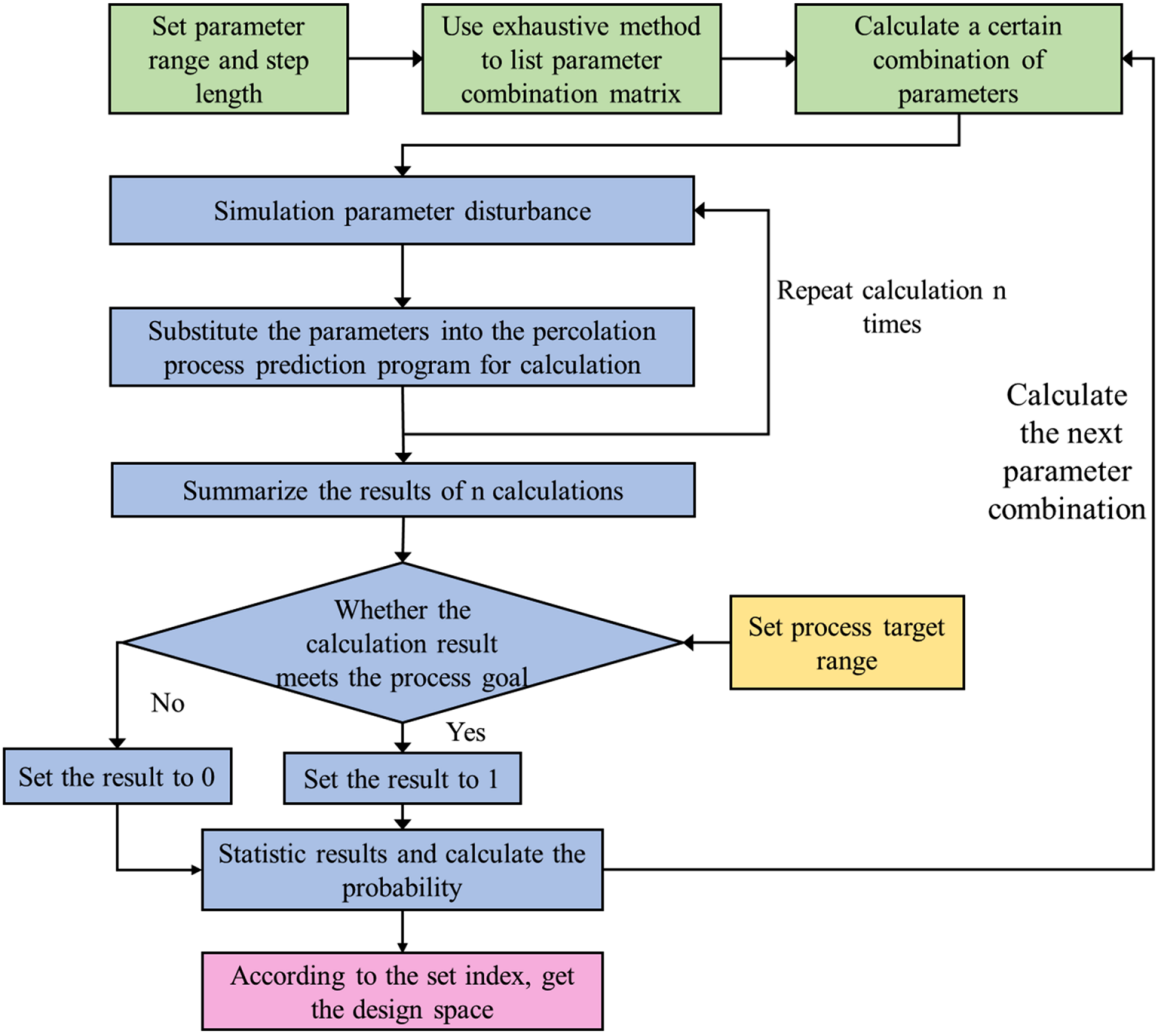
Design space calculation flow chart.

Matlab software (MathWorks, 2018b) was used to write the program and calculate the design space. The calculation flow chart was shown in Fig. 3. The Design space calculations took parameter perturbations into account. Before substituting a parameter combination into the percolation formula for calculation, a random value within a certain range was added to the set optimized parameter value to simulate the parameter disturbance generated in actual production. After calculating the parameter combination n times, the obtained result can be considered as the result group that may be produced by the parameter combination under different errors. Comparing the values in the result group with the set target range, the probability of the results that can reach the target in the result group can be obtained, that is, the probability of reaching the target. Different parameter combinations were calculated to obtain the probability of reaching the standard of each parameter combination, and the range of parameter combinations higher than the set probability of reaching the standard is regarded as the design space.

## Results and discussion

### Kinetics of impregnation extraction

**Figure 4:**
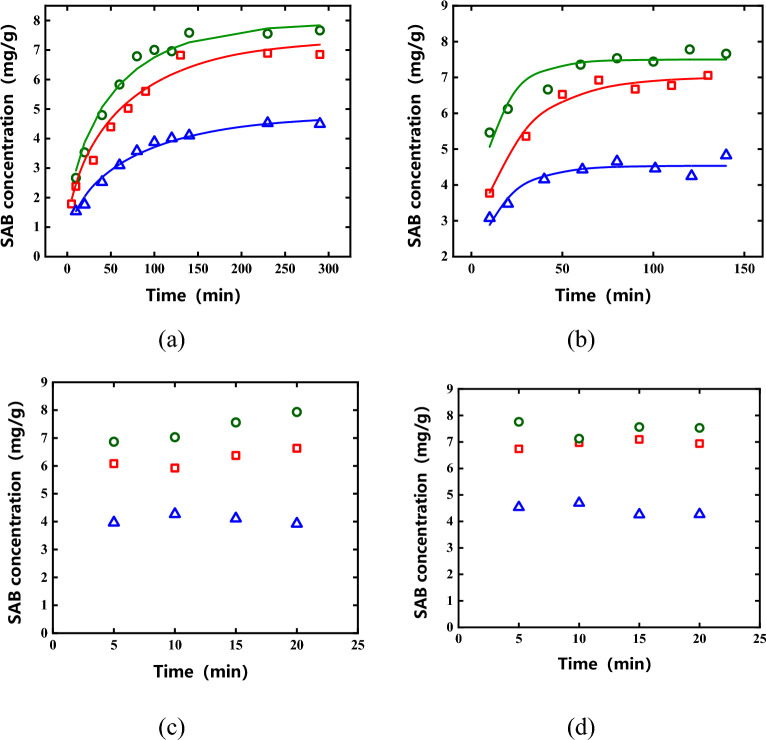
Impregnation kinetic curve. (**a**) 5–10 mesh; (**b**) 10–24 mesh; (**c**) 24–50 mesh; (**d**) 50–65 mesh (open red square represents D1, open green circle represents D2, open blue triangle represents D3, — represents Diffusion model fit curve).

The impregnation kinetic curves of *Salvia miltiorrhiza* with four particle sizes of 5–10, 10–24, 24–50, and 50–65 mesh in three batches of D1, D2, and D3 were measured. The impregnation kinetic curves were shown in Fig. 4. It can be seen from Fig. 4c and (d) that when the particle size of the *Salvia miltiorrhiza* medicinal pieces was small, equilibrium of impregnation was reached within 5 min. This may be due to the following two reasons. Firstly, when the medicinal material powders were smaller, more cell walls were destroyed in the pulverization process, which reduced the mass transfer resistance. Secondly, the smaller particle size of the medicinal material increased its surface area for mass transfer. At the same time, the specific surface area of the medicinal material increased, which was also conducive to the diffusion of the active ingredient. It can be seen from Fig. 4a,b that the concentration of SAB increased at first when the particle size of the salvia medicinal pieces was larger, then gradually tended to reach equilibrium after a certain period of time. The content of active ingredients in medicinal materials from different sources was quite different, and therefore the concentration of the extract was also different when they were in equilibrium. On the whole, the impregnation equilibrium can be achieved in a relatively short period of time, and even 5–10 mesh medicinal materials can basically reach the impregnation equilibrium within 5 h.

Since the 24–50 mesh and 50–65 mesh medicinal materials balance quickly, only the 5–10 mesh and 10–24 mesh medicinal materials were used for kinetic model fitting. The kinetic model fitting results of the three batches of medicinal materials were shown in Table 3. It can be seen from Table 3 that for the impregnation curves of *Salvia miltiorrhiza* with different batches and different particle sizes, the fitting effect of each kinetic model was relatively good, and the R^2^ obtained by most fittings was greater than 0.90, among which the Diffusion model had the best fitting effect. In fact, we can find that the average R^2^ of Peleg's model and the Diffusion model is relatively close, indicating that both models can fit kinetic curves well. However, considering that the physical meaning of the Diffusion model parameters is more explicit (where ${D}_{eff}$ is the apparent diffusion coefficient), the Diffusion model was finally chosen. The curve obtained from the fitting was shown in Fig. 4, and the parameter values obtained from the fitting were shown in Table 4. It can be seen that the fitting parameters of the same batch of medicinal materials of different particle sizes were relatively close, which indicated that the batch of medicinal materials is an important factor affecting the maceration process.

**Table 3:** R^2^ obtained by fitting different impregnation models.

Numbers of Salvia	Particle size (mesh number of sieve)	First order kinetic model	Peleg’s model	Diffusion model
D1	5–10	0.9146	0.9300	0.9509
	10–24	0.9296	0.9669	0.9752
D2	5–10	0.9624	0.9780	0.9769
	10–24	0.8676	0.9735	0.9484
D3	5–10	0.9485	0.9765	0.9840
	10–24	0.8090	0.8991	0.8952
Average value		0.9053	0.9540	0.9551

**Table 4:** Parameter values from the diffusion model fit.

Numbers of Salvia	Particle size (mesh number of sieve)	*C*_*eq*_(mg/g)	$\frac{{D}_{eff}}{{r}^{2}}$ (min^−1^)
D1	5–10	7.366	0.00112
	10–24	7.017	0.00366
D2	5–10	7.916	0.00144
	10–24	7.565	0.00698
D3	5–10	4.788	0.00102
	10–24	4.535	0.00567

### Percolation curve

**Figure 5:**
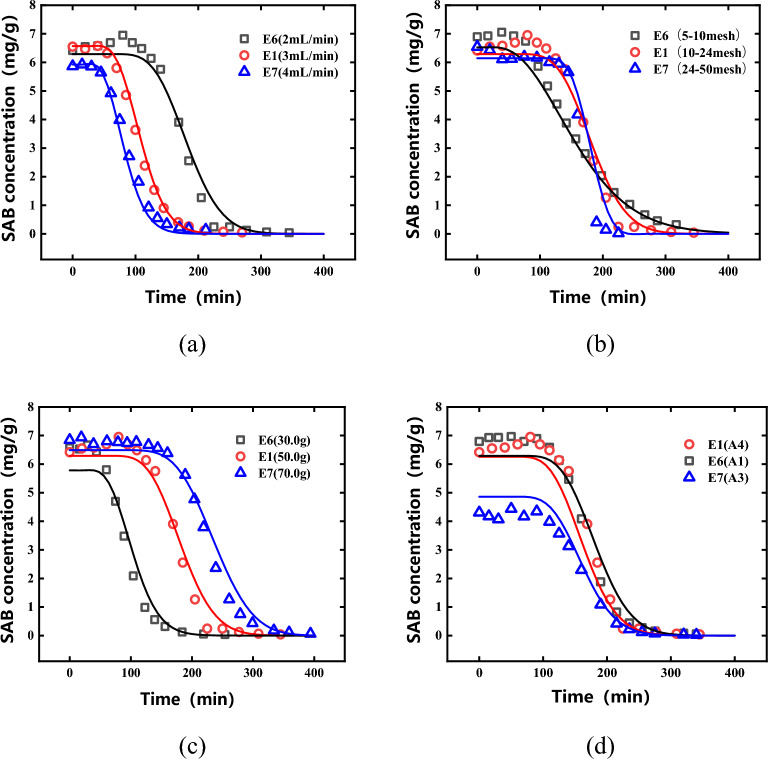
The percolation curve of *Salvia miltiorrhiza* under different conditions. (**a**) Percolation flow; (**b**) Piece size; (**c**) Dosage of medicinal materials; (**d**) batch of medicinal materials (point represents experimental value, — represents predictive value).

According to Table 2, the obtained single-factor percolation experiment results were shown in Fig. 5. It can be seen from Fig. 5 that when the particle size was larger, the diffusion resistance of the components in the medicinal particles was relatively large. Therefore, the diffusion rate of SAB was slow, the concentration of the percolation curve decreased more slowly, and the time required to achieve the same target yield was longer. When the percolation flow rate was larger, the concentration of percolation curve decreases faster, and the extraction speed was higher. The mass of the medicinal material mainly affected the final SAB yield but had no obvious effect on the decreasing speed of the percolation curve. For different batches of *Salvia miltiorrhiza*, the higher the SAB content per unit mass, the higher the final yield. This showed that the percolation process of *Salvia miltiorrhiza* was affected by multiple factors.

### Determination of SAB content in medicinal materials

In this work, the content of SAB in *Salvia miltiorrhiza* was determined by ultrasonic extraction and multiple impregnation methods. The content of SAB in some batches of medicinal materials measured by ultrasonic extraction method and multiple dipping method was shown in Table 5. The results of ultrasonic extraction method and multiple impregnation method had little difference. Compared with the multiple impregnation method, the ultrasonic extraction method was simpler and less time-consuming. Therefore, the content of SAB in *Salvia miltiorrhiza* was determined by ultrasonic extraction method. From the measurement results, the content of SAB in different batches of medicinal materials varies greatly, but the particle size had little effect on the maximum extraction amount of SAB. This suggests that if the extraction time is longer, we can still achieve a satisfactory extraction effect without using smaller particle sizes.

**Table 5:** The maximum extraction amount of SAB in *Salvia miltiorrhiza.*

Numbers of *Salvia miltiorrhiza*	Particle size (mesh number of sieve)	SAB content in *Salvia miltiorrhiza* (mg/g)	
		Multiple impregnation	Ultrasonic extraction
D1	10–24	45.80 ± 0.02	43.83 ± 0.42
D2	10–24	52.23 ± 0.01	51.27 ± 1.21
D4	5–10	51.63 ± 0.02	49.60 ± 0.42
	10–24	50.45 ± 0.02	
	24–50	49.39 ± 0.01	

### Determination of ***D***_***is***_ of medicinal materials

According to Formula (3), the* D*_*is*_ of different batches of *Salvia miltiorrhiza* with different particle sizes were calculated as shown in Table 6. It can be seen from Table 6 that the *D*_*is*_ of SAB in different batches and different particle sizes of *Salvia miltiorrhiza* in the immersion stage were similar, basically between 1.1 and 1.35. There was a certain gap in the *D*_*is*_ of medicinal materials between different batches, but the average value is between 1.15 and 1.30. Among different particle sizes in the same batch, the *D*_*is*_ increased slightly with the decrease of particle size. Under the same particle size in the same batch, the overall difference of the *D*_*is*_ of medicinal materials measured by different solid–liquid ratios was small. Due to the small change of the *D*_*is*_, in order to simplify the model parameters, the average value of the *D*_*is*_ of each batch of 10–24 mesh medicinal materials was 1.21 to predict the *D*_*is*_ value of the subsequent percolation process.

**Table 6:** *D*_*is*_ measurement results.

Numbers of Salvia		Solid–liquid ratio	Ultrasonic extraction	Average value of *D*_*is*_
D1	10–24	1:6	1.16	1.22
		1:8	1.32	
		1:10	1.17	
D2	10–24	1:6	1.18	1.15
		1:8	1.10	
		1:9	1.17	
D4	5–10	1:6	1.24	1.25
		1:7	1.30	
		1:8	1.23	
		1:10	1.23	
	10–24	1:6	1.26	1.28
		1:7	1.24	
		1:8	1.31	
		1:10	1.30	
	24–50	1:6	1.26	1.29
		1:7	1.23	
		1:8	1.36	
		1:10	1.30	

### Determination of SAB concentration at the initial time of percolation

According to the impregnation kinetic data in 3.1, the time to reach equilibrium was relatively short during the impregnation of *Salvia miltiorrhiza*, and the initial time of dynamic percolation can be regarded as the impregnation equilibrium time. Therefore, Formula (15) can be derived and calculated according to Formulas (4) and (5). The predicted value ${C}_{w}^{0}$ at the initial moment of the single-factor experiment of percolation was obtained. At the same time, the relative error *RE* of its prediction was calculated according to Formula (16).

15 $${C}_{w}^{0}=\frac{{M}_{0}m}{{D}_{is}{V}_{s}+{V}_{w}}$$

16 $$RE=\frac{\left|\widehat{{y}_{i}}-{y}_{i}\right|}{{y}_{i}}\times 100\mathrm{\%}$$

**Figure 6:**
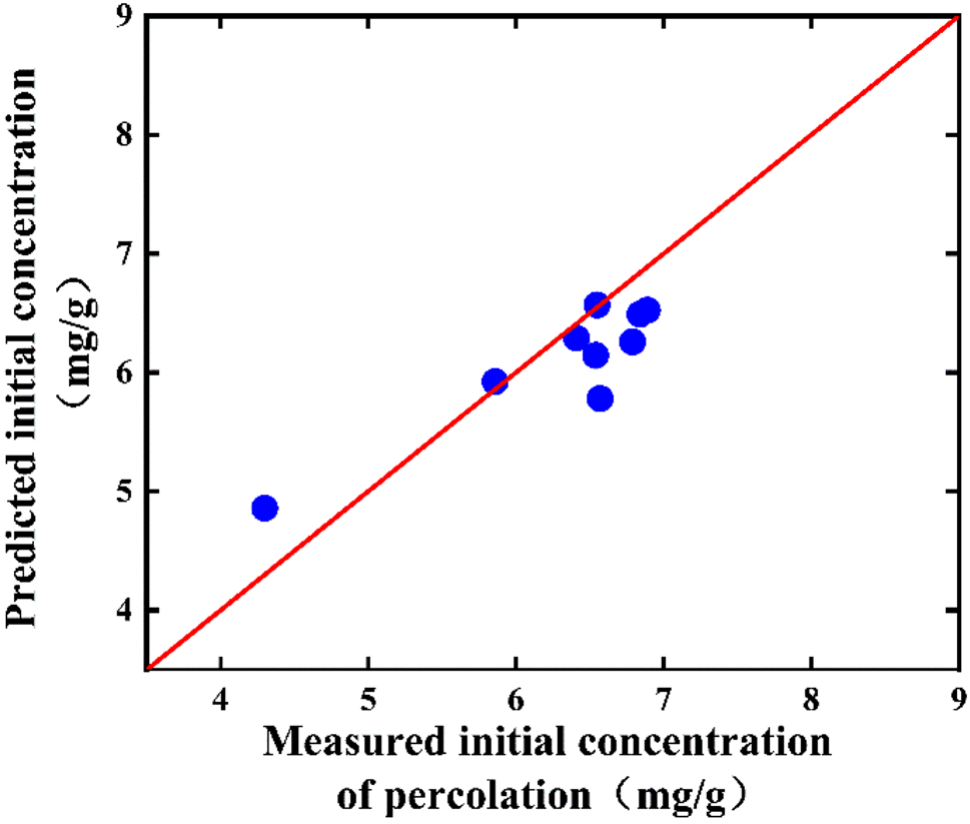
Predicted and experimental values of outlet concentration at the initial time of single-factor percolation experiment.

The experimental value and predicted value at the initial time of the single-factor percolation experiment predicted by Formula (15) were shown in Fig. 6, and *RE* value was 5.8%. Overall, the predicted value of the SAB concentration at the initial time of percolation was not much different from the actual value, and the relative error was less than 10%, indicating that the prediction method was feasible. Although the medicinal materials were immersed in the percolation column and could not be shaken like the immersion kinetics test, the solid–liquid equilibrium was basically achieved due to the long immersion time.

### Determining the particle size of the medicinal material, the bed layer voidage , and the expansion coefficient of the medicinal material

In this study, different types of sieves were used to distinguish the particle size of the Chinese medicinal powder. Therefore, according to the size of the mesh, the average value of the mesh diameter of the mesh was taken as the particle size of the medicinal material, which were 0.3 cm (5–10 mesh), 0.1425 cm (10–24 mesh), 0.0603 cm (24–50 mesh). After the percolation, the water in percolation column was obtained by filtration, the volume was measured, and the ratio of the water to the total volume of the percolation column was regarded as the bed layer voidage. When predicting the percolation curve, the total height* H* of the percolation column needs to be known. Therefore, according to the nine groups of single factor experiments in Table 2, the expansion coefficient *α* of the medicinal material (the volume of the medicinal material per unit weight after the medicinal material was fully swelled) was calculated. The volume in the percolation cylinder after swelling, and then roughly predicts the total height *H* of the percolation column during percolation. Among them, the expansion coefficient of medicinal materials is calculated by Formula (17).

17 $$\alpha =\frac{\pi {d}^{2}h\varepsilon }{4m}$$

where, *d* represents diameter of seepage tube. *H* was calculated by Formula (18).

18 $$H=\frac{4m\alpha }{(1-\varepsilon )\pi {d}^{2}}$$

The *ε* and α of the medicinal materials measured in the single-factor percolation experiment obtained according to Table 2 were shown in Table 7. It can be seen from Table 7 that under different batches and experimental conditions, the *ε* was not much different, which was around 0.3–0.4, and the *α* is mostly between 3.5 and 4.5. In order to simplify the calculation, both the voidage and the expansion ratio coefficient of medicinal materials were taken as average values, which were 0.37 and 3.81, respectively, and were set as fixed values in the subsequent prediction calculation.

**Table 7:** *ε* and* α* measured by single factor percolation experiment.

Experiment	The average particle size (μm)	*ε*	*α* (mL/g)
E1	1425	0.41	3.89
E2	1425	0.35	4.08
E3	1425	0.37	3.80
E4	1425	0.36	3.80
E5	1425	0.34	3.97
E6	3000	0.41	3.48
E7	603	0.30	4.40
E8	1425	0.41	3.43
E9	1425	0.42	3.42
Average value		0.37	3.81

### Determination of the calculation formulas of mass transfer coefficient and axial diffusion coefficient

From the analysis in Fig. 2, it can be seen that the leaching of components in *Salvia miltiorrhiza* mainly includes two steps, one was the mass transfer from the inside of the medicinal material to the surface of the medicinal material, and the other was diffusion from the medicinal material surface to the solution. Therefore, when calculating, the mass transfer coefficient (*K*_*x*_) can be divided into internal mass transfer coefficient (*k*_*int*_) and external mass transfer coefficient (*k*_*ext*_*)*. The calculation formula was listed as Formula (19)^16^.

19 $${K}_{x}={ \left[\frac{1}{{k}_{int}}+\frac{1}{{k}_{ext}}\right]}^{-1}$$

The formula for calculating the internal mass transfer coefficient was shown in Formula (20)^16^. The value of *D*_*eff*_ can be calculated according to the $\frac{{D}_{eff}}{{r}^{2}}$ value of *Salvia miltiorrhiza* dipping by fitting. The average value of *D*_*eff*_ in 5–10 mesh was 1.073 × 10^–8^ m^2^/min, and the average value of *D*_*eff*_ in 10–24 mesh was 1.104 × 10^–8^ m^2^/min. The two values were similar, and because of the simplicity of the calculation, the average can be taken 1.089 × 10^–8^ m^2^/min.

20 $${k}_{int}=\frac{5{D}_{eff}}{r}$$

There were many reports about the external mass transfer coefficient. Several widely used mass transfer coefficient formulas shown in Table 8 were selected for trial fitting, and the more suitable external mass transfer coefficient formula was selected from them.

**Table 8:** *k*_*ext*_ calculation model.

Author	Formula	References
Vermeulen	${k}_{ext}=\frac{5.21{\left({D}_{m}{u}_{0}\right)}^{0.5}r}{{6(2r)}^{1.5}\left(1-\varepsilon \right)}$	^17^
Williamson	${k}_{ext}=2.4{u}_{0}{\left(Re\right)}^{-0.66}{\left(Sc\right)}^{-0.58}$	^18^
Wilson and Geankoplis	$Sh=\frac{1.09}{\varepsilon }{\left(Re\right)}^{1/3}{\left(Sc\right)}^{1/3}$	^19^
Ranz and Marshall	$Sh=2+0.6{\left(Sc\right)}^{1/3}{\left(Re\right)}^{1/2}$	^20^
Wakao and Funazkri	$Sh=2+1.1{\left(Sc\right)}^{1/3}{\left(Re\right)}^{0.6}$	^21^

In the table, *Sh* was the Sherwood number, which was calculated by Formula (21). *Sc* was the Schmidt number, which was calculated by Formula (22). *Re* was the Reynolds number, which was calculated by Formula (23).

21 $$Sh=\frac{2{k}_{ext}r}{{D}_{m}}$$

22 $$Sc=\frac{\mu }{\rho {D}_{m}}$$

here, *ρ* was solution density, *μ* was viscosity coefficient.

23 $${R}_{e}=\frac{2\rho {u}_{0}r}{\mu }$$

*D*_*m*_ was molecular diffusion coefficient of solute, the calculation method of which can be seen in supplementary material. The calculation result was 5.83 × 10^–10^ m^2^/s.

The Matlab was used for calculation, and the calculation results of nine groups of single-factor percolation experiments using different calculation formulas for *K*_*ext*_ were shown in Table 9.

**Table 9:** Calculation results of different *K*_*ext*_ calculation formulas (m/min).

Experiment number	Calculation formulas				
	Vermeulen	Williamson	Wilson and Geankoplis	Ranz and Marshall	Wakao and Funazkri
E1	1.82 × 10^–4^	7.00 × 10^–4^	2.20 × 10^–3^	1.18 × 10^–4^	7.00 × 10^–5^
E2	1.79 × 10^–4^	7.41 × 10^–4^	3.02 × 10^–3^	1.21 × 10^–4^	7.20 × 10^–5^
E3	1.79 × 10^–4^	7.27 × 10^–4^	2.70 × 10^–3^	1.20 × 10^–4^	7.13 × 10^–5^
E4	2.19 × 10^–4^	8.42 × 10^–4^	4.28 × 10^–3^	1.31 × 10^–4^	7.79 × 10^–5^
E5	2.53 × 10^–4^	9.47 × 10^–4^	6.40 × 10^–3^	1.41 × 10^–4^	8.45 × 10^–5^
E6	1.25 × 10^–4^	4.30 × 10^–4^	2.20 × 10^–3^	6.51 × 10^–5^	3.88 × 10^–5^
E7	2.76 × 10^–4^	1.38 × 10^–3^	4.11 × 10^–3^	2.51 × 10^–4^	1.52 × 10^–4^
E8	1.81 × 10^–4^	7.14 × 10^–4^	2.43 × 10^–3^	1.19 × 10^–4^	7.06 × 10^–5^
E9	1.83 × 10^4^	6.96 × 10^4^	2.10 × 10^–3^	1.17 × 10^4^	6.97 × 10^–5^

As shown in Table 9, the mass transfer coefficient values calculated by different formulas were quite different, and the order of magnitude spans from 10^–3^ to 10^–5^ m/min. Among them, the Wilson and Geankoplis formula calculated the largest value, and the Wakao and Funazkri formula calculated the smallest value. When the nine groups of single factor experiments were calculated with the same formula, there was no significant difference in the experimental values of the five groups E1–E3, E8, and E9, which proved that the batch of medicinal materials and the quality of medicinal materials had no great influence on the *K*_*ext*_. The experimental results of E1, E4 and E5 groups showed that the value of *K*_*ext*_ increases with the increase of percolation flow rate. The experimental results of E1, E6 and E7 showed that the *K*_*ext*_ increased with the decrease of particle size. It was proved that the percolation flowrate and the particle size of the medicinal pieces were the main factors affecting the *K*_*ext*_. The calculation of the *D*_*ax*_ was fitted with the formula in Table 10.

**Table 10:** The calculation formula of *D*_*ax*_*.*

Authors	Calculation formula	Reference
Chung and Wen	${D}_{ax}=\frac{2R\varepsilon {u}_{0}}{0.2+0.011{{R}_{e}}^{0.48}}$	^22^
Athayle	${D}_{ax}=2{u}_{0}r{\left(\frac{{P}_{e}}{1-\varepsilon }\right)}^\frac{1}{6}$	^23^
Koch and Brady	${D}_{ax}={2ru}_{0}\varepsilon \left[\frac{3}{4}+\frac{{\pi }^{2}}{6}\left(1-{\varepsilon }_{b}\right)\mathrm{ln}\left({P}_{e}\right)+\frac{1}{{P}_{e}}\right]$	^24^
Gunn	$\frac{{D}_{ax}}{{u}_{0}r}=2Z{(1-p)}^{2}-{Z}^{2}p{\left(1-p\right)}^{3}\left[1-\mathrm{exp}\left(\frac{-1}{Zp\left(1-p\right)}\right)\right]+\frac{\varepsilon }{1.4Pe}$ $Z=\frac{Pe}{23.1361(1-\varepsilon )}\,p=0.17+0.33exp(-\frac{24}{Re})$	^25^
Ruthven	${D}_{ax}=0.5{D}_{m}+1.4r{u}_{0}$	^26^

Where, the calculations formula of Peclet number (*Pe*) was shown in Formula (24).

24 $$Pe=Re\cdot Sc$$

The calculation results of nine groups of single-factor percolation experiments using different axial diffusion coefficient calculation formulas were shown in Table 11.

**Table 11:** *D*_*ax*_ results from calculation formula (m^2^/min).

Experiment number	Calculation formulas				
	Chung and Wen	Athayle	Koch and Brady	Gunn	Ruthven
E1	7.24 × 10^–6^	3.91 × 10^–4^	4.89 × 10^–6^	6.45 × 10^–6^	4.96 × 10^–6^
E2	7.24 × 10^–6^	5.28 × 10^–4^	5.57 × 10^–6^	7.67 × 10^–6^	5.81 × 10^–6^
E3	7.24 × 10^–6^	4.75 × 10^–4^	5.34 × 10^–6^	7.22 × 10^–6^	5.49 × 10^–6^
E4	1.09 × 10^–5^	1.13 × 10^–3^	8.87 × 10^–6^	1.22 × 10^–5^	8.47 × 10^–6^
E5	1.45 × 10^–5^	2.23 × 10^–3^	1.30 × 10^–5^	1.81 × 10^–5^	1.20 × 10^–5^
E6	1.52 × 10^–5^	1.73 × 10^–3^	1.19 × 10^–5^	1.57 × 10^–5^	1.04 × 10^–5^
E7	3.06 × 10^–6^	1.27 × 10^–4^	2.17 × 10^–6^	2.87 × 10^–6^	2.86 × 10^–6^
E8	7.24 × 10^–6^	4.30 × 10^–4^	5.11 × 10^–6^	6.81 × 10^–6^	5.21 × 10^–6^
E9	7.24 × 10^–6^	3.74 × 10^–4^	4.79 × 10^–6^	6.28 × 10^–6^	4.84 × 10^–6^

It can be seen from Table 11 that the *D*_*ax*_ calculated by the Athayle formula was larger, and the calculation results of the other four formulas were similar, all within the order of magnitude of 10^–5^–10^–6^. From the calculation results obtained by nine groups of single factor experiments using the same formula, the *D*_*ax*_ values of the five groups of E1, E2, E3, E8, and E9 were similar, indicating that the quality of the medicinal material and the batch of medicinal materials had different *D*_*ax*_, whose effect was relatively small. However, the *D*_*ax*_ values of the three groups of experiments E1, E4, and E5 were different. With the increase of percolation flow rate, the *D*_*ax*_ increased. The *D*_*ax*_ values of the three groups of experiments E1, E6 and E7 increased with the increase of the particle size of the medicinal pieces. To sum up, it showed that the percolation flowrate and the particle size of the medicinal pieces had a relatively large influence on the *D*_*ax*_.

In order to further screen the appropriate *k*_*ext*_ and *D*_*ax*_, the formulas of different* K*_*ext*_ and *D*_*ax*_ were combined in pairs and substituted into nine groups of single-factor percolation experiments for prediction. The average R^2^ obtained in nine groups of experiments for different formula combinations was shown in Table 12.

**Table 12:** The *K*_*ext*_ and *D*_*ax*_ formulas fitted the average R^2^ obtained from single-factor experiments.

*D*_*ax*_	*k*_*int*_				
	Vermeulen	Williamson	Wilson and Geankoplis	Ranz and Marshall	Wakao and Funazkri
Chung and Wen	0.9674	0.9701	0.9704	0.9650	0.9565
Athayle	0.7114	0.7148	0.7154	0.7084	0.7001
Koch and Brady	0.9697	0.9716	**0.9718**	0.9676	0.9598
Gunn	0.9676	0.9703	0.9706	0.9651	0.9566
Tan and Liou	0.9695	0.9714	0.9716	0.9675	0.9598

Significant values are in bold.

As shown in Table 12, the fitted result R^2^ obtained in the table was the average value of the fitted R^2^ of nine groups of single-factor percolation experiments. The combination of Wilson and Geankoplis and Koch and Brady formula were more suitable for the *k*_*int*_ in the *Salvia miltiorrhiza* percolation experiment and calculation of the* D*_*ax*_.

The calculation results of the above various parameters were substituted into nine groups of *Salvia miltiorrhiza* single-factor percolation experiments. The results were shown in Fig. 5. The predicted curves were similar to the actual curves, and R^2^ was greater than 0.94, which proved that the established mechanism model had a good prediction effect and was more reliable.

### Sensitivity analysis of seepage parameters

In order to measure the influence of measurement errors of different parameters on the final prediction effect, the sensitivity analysis of percolation parameters was carried out in this study, including *r*, *ε*, *D*_*is*_, *k*_*int*_, *k*_*ext*_, *D*_*ax*_. Taking the parameter values obtained from the E1 percolation experiment as an example, the measured values of different parameters and the set error ranges were shown in Table 13. Sensitivity analysis was carried out with Matlab, and 10,000 simulations were performed on the percolation process of *Salvia miltiorrhiza*. During the simulation, the measurement or calculation errors within the range shown in Table 13 were randomly generated, and the percolation process of *Salvia miltiorrhiza* was predicted according to these random experimental errors.

**Table 13:** Measured values of different parameters and set disturbance range.

Pparameter	Estimated value	Set error range
*ε*	0.41	± 0.05
*r*(m)	1.425 × 10^–3^	± 0.5 × 10^–3^
*D*_*is*_	1.21	± 0.10
*k*_*int*_ (m/min)	7.64 × 10^–5^	± 1.00 × 10^–5^
*k*_*ext*_ (m/min)	2.20 × 10^–3^	± 1.00 × 10^–3^
*D*_*ax*_ (m^2^/min)	4.89 × 10^–6^	± 1.00 × 10^–6^

**Figure 7:**
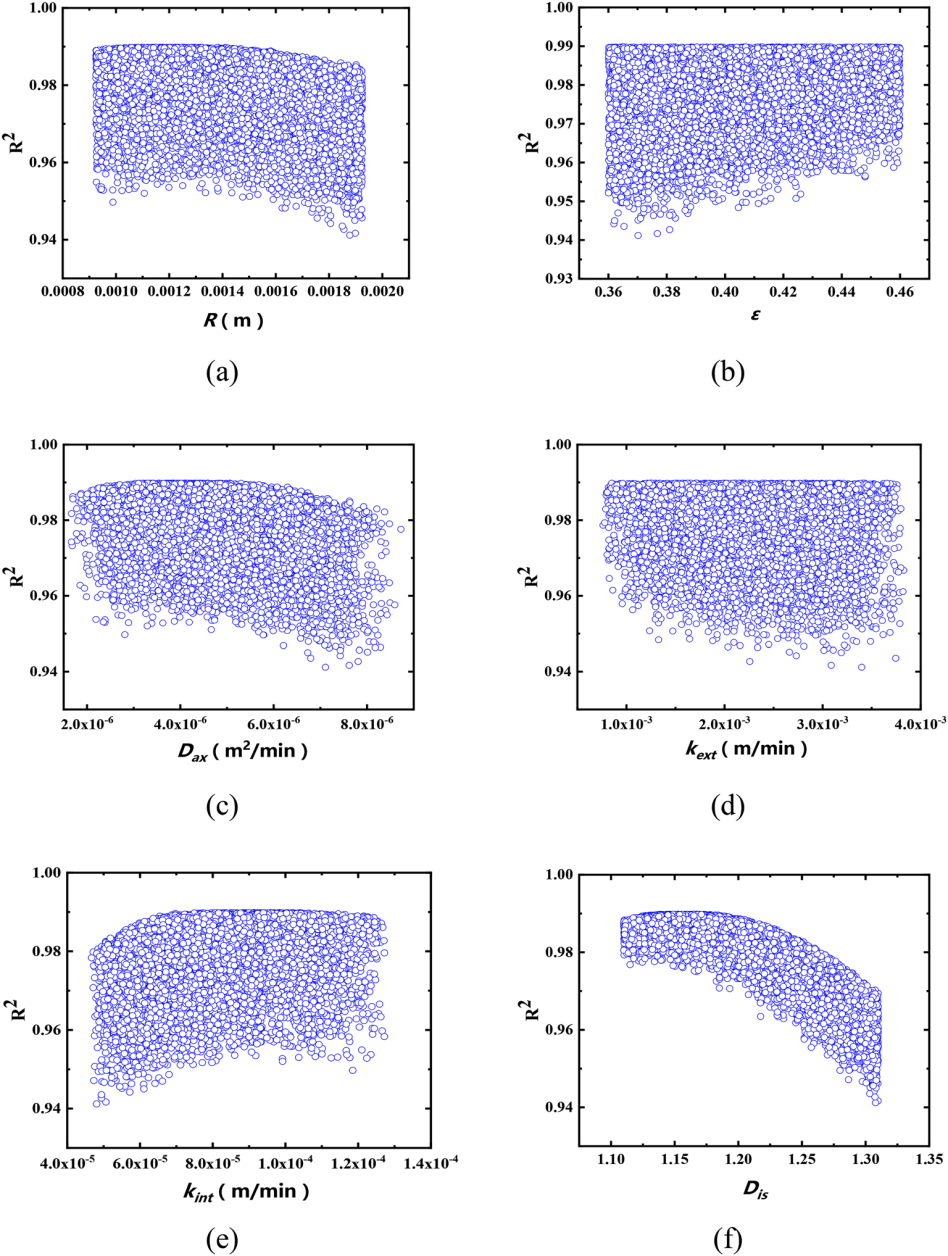
Sensitivity analysis results.

The scatter diagram between each process parameter and R^2^ were shown in Fig. 7, and the correlation coefficient values were shown in Table 14. In Fig. 7a–f, the distribution law of the scatter points in Fig. 7f was the most obvious. The upper edge of the scatter diagram was smooth, indicating that the *D*_*is*_ had the greatest influence on R^2^. When the *D*_*is*_ was between 1.0 and 1.2, R^2^ was the highest and the variation range was small. When the *D*_*is*_ was between 1.2 and 1.3, R^2^ decreased with the increase of the *D*_*is*_.

**Table 14:** Correlation coefficient values of different parameters and R^2^.

Parameter	*ε*	*r*	*k*_*int*_	*k*_*ext*_	*D*_*ax*_	*D*_*is*_
Relative coefficient	0.182	− 0.179	0.246	− 0.014	0.157	− 0.827
*P* value	0.000	0.000	0.000	0.000	0.000	0.000

It can be seen from Table 14 that the correlation coefficients between the five parameters of *r*, *ε*, *D*_*is*_, *k*_*int*_, *k*_*ext*_, *D*_*ax*_ and R^2^ of the fitted results were all significant (*P* value < 0.01). The correlation coefficients between the three parameters of *ε*, *k*_*ext*_, *D*_*is*_ and R^2^ were all negative numbers, indicating that when the measured values of *ε*, *k*_*ext*_, *D*_*is*_ were smaller, R^2^ was larger. The correlation coefficient between the three parameters of *r*, *D*_*ax*_, *k*_*int*_ and R^2^ was a positive value, indicating that when the measured values of *r*, *D*_*ax*_, *k*_*int*_ were larger, R^2^ was larger. Among the six parameters, the absolute values of the relative coefficients of *r*, *ε*, *k*_*int*_, *k*_*ext*_, *D*_*ax*_ were all between 0.1 and 0.2, while the absolute value of the relative coefficient of the *D*_*is*_ reached 0.8, indicating that the *D*_*is*_ had a great influence on R^2^.

### Calculation of design space

**Figure 8:**
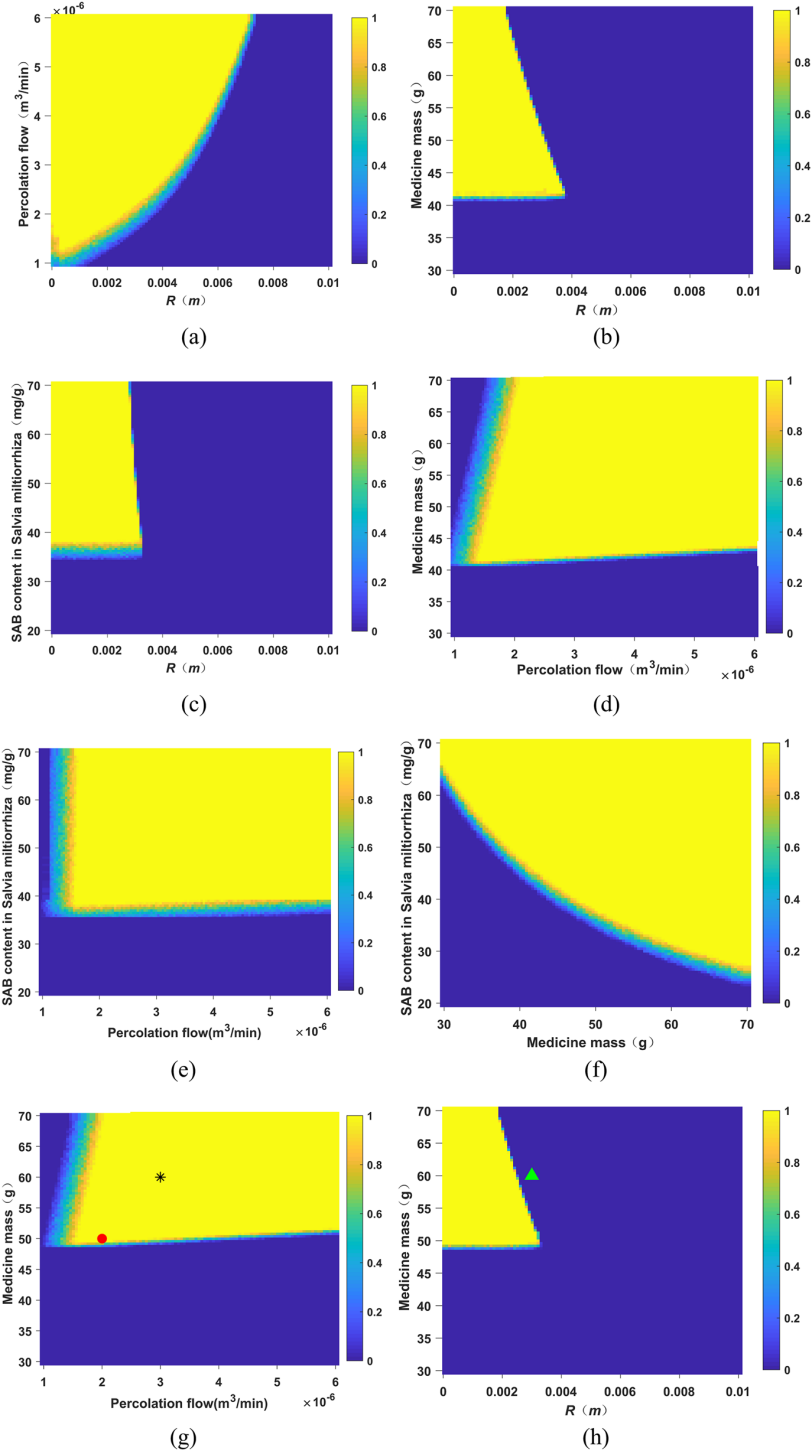
Design space diagram. (**a**) Medicine mass = 50.0 g; SAB content in *Salvia miltiorrhiza* = 45 mg/g. (**b**) Percolation flow = 2 mL/min; SAB content in *Salvia miltiorrhiza* = 45 mg/g. (**c**) Percolation flow = 2 mL/min; medicine mass = 50.0 g. (**d**) *R* = 0.0015 m; SAB content in *Salvia miltiorrhiza* = 45 mg/g. (**e**) *R* = 0.0015 m; medicine mass = 50.0 g. (**f**) Percolation flow = 2 mL/min; *R* = 0.0015 m. (**h**) *R* = 0.001425 m; SAB content in *Salvia miltiorrhiza* = 37.28 mg/g. (**g**) Percolation flow = 2 mL/min; SAB content in *Salvia miltiorrhiza* = 37.28 mg/g (different colors in the figure represented different probability of reaching the standard, and the color bar on the right represents the corresponding probability of reaching the standard, filled red circle was the value of experiment 1, * was the value of experiment 2, filled green triangle was the value of experiment 3).

The design space was calculated. The evaluation index was set as the final concentration of percolation was less than 0.1 mg/g, and the yield of SAB was greater than 1700 mg. According to the previous experimental results, some experimental parameters were fixed in the design space calculation, the expansion coefficient of medicinal materials was fixed at 3.81, the bed layer voidage was fixed at 0.37, and the volume partition coefficient was fixed at 1.21. The properties of the medicinal materials, the range of parameters, and the range of parameter disturbance during the calculation are shown in Table 15. Each experimental point was repeatedly calculated 100 times to calculate the probability of reaching the standard. When the probability of reaching the standard exceeds 0.9, the combination of properties and parameters of the medicinal material is considered to be within the design space. The design space was calculated according to the properties of medicinal materials (particle size of medicinal pieces and content of SAB) and process parameters (percolation flow rate, dosage of medicinal materials), as shown in Fig. 8.

**Table 15:** Parameter range and disturbance range.

Parameter	Range	Disturbance range
*r* (m)	0.01–0.0001	0.0001
Percolation flow (mL/min)	1.0–6.0	0.3
Medicine mass (g)	30.0–70.0	0.5
SAB content in *Salvia miltiorrhiza* (mg/g)	20.0–70.0	1.0

In order to verify the reliability of the design space, points were selected inside and outside the design space for verification, and the new batch A5 was selected for the experiment. The specific verification experimental conditions are shown in Table 16 and Fig. 8g–h, and the results were shown in Table 17. The results showed that the measured values obtained by the three sets of verification experiments were close to the predicted values, indicating that the model had good predictability and the built design space was more reliable.

**Table 16:** Design space validation experiment point conditions (n = 3).

Verify conditions	Experiment 1	Experiment 2	Experiment 3
Is it within the design space	Yes	Yes	No
Percolation flow (mL/min)	2	3	2
*r* (m)	0.001425	0.001425	0.003
Medicine mass (g)	50.0	60.0	60.0
SAB content in *Salvia miltiorrhiza* (mg/g)	37.28	37.28	37.28

**Table 17:** Design space validation experiment point results (n = 3).

Verify conditions		Experiment 1	Experiment 2	Experiment 3
SAB production (mg)	Measured value ± SD	1769 ± 43.01	2122 ± 30.38	2203 ± 47.81
	Predicted value	1723	2100	2100
endpoint concentration (mg/g)	Measured value ± SD	0.0150 ± 0.00576	0.00750 ± 0.00208	0.356 ± 0.0993
	Predicted value	1.44 × 10^–4^	9.79 × 10^–7^	0.162

## Model application

### Quantitative extraction

The quality of Chinese herbal medicines often varies greatly between batches. In order to stabilize the quality of proprietary Chinese medicines, it is sometimes necessary to control the extraction amount within a range. In order to verify the feasibility of quantitative extraction using the established mechanism model, the D5 batch of *Salvia miltiorrhiza* with a particle size of 0.001425 m and a SAB content of 37.28 mg/g in *Salvia miltiorrhiza* was selected as the percolating medicinal material. The target yield is shown in Table 18. Two validation experiments were carried out, V1 and V2, respectively.

**Table 18:** Quantitative extraction and verification of experimental results.

Verify conditions	V1	V2
Target yield (mg)	1700–1730	2060–2090
Forecast end time range (min)	216–247.5	180–207
Actual end time (min)	223	187
Measured yield value (mg)	1721	2085

Experiment V1 used 50.0 g medicinal materials, the particle size was 0.001425 m, and the percolation flow rate was 2 mL/min. Experiment V2 used 60.0 g medicinal materials with a particle size of 0.001425 m and a flow rate of 3 mL/min. The verification results are shown in Table 18. The measured value of the verification experiment is close to the predicted value, indicating that the model has good predictability, and the model is more reliable to predict the end point of percolation.

### Endpoint prediction

During the percolation production process of traditional Chinese medicine, besides optimizing the process parameters to obtain better product quality, the determination of when to end the percolation (endpoint) is also particularly important. Incomplete, resulting in waste of medicinal materials. Ending percolation too late may result in wasted solvent and time. Taking the concentration of SAB in the leachate less than 0.1 mg/g as the standard, the established mechanism model was used to predict the endpoint, and two groups of experiments V3 and V4 were selected for verification. The D5 batch of *Salvia miltiorrhiza* with the content of SAB of 37.28 mg/g was used as the osmotic medicinal material in both groups of experiments. Experiment V3 used 50.0 g medicinal materials, the particle size of the medicinal pieces was 0.001425 m, and the percolation flow rate was 2 mL/min. Experiment V4 used 60.0 g of medicinal materials, the particle size of the medicinal pieces was 0.001425 m, and the percolation flow rate was 3 mL/min. The verified experimental results are shown in Table 19. The endpoint prediction and validation experiments showed that the measured values were close to the predicted values, indicating that the model had good predictability and the model was more reliable in predicting the end point of percolation.

**Table 19:** Endpoint prediction validation experimental results.

Verify conditions	V3	V4
Target endpoint concentration (mg/g)	< 0.1	< 0.1
Forecast end time range (min)	> 276	> 225
Actual end time (min)	285	226
Measured value of end point concentration (mg/mL)	0.0815	0.0949

## Conclusion

In this work, a mechanism model of percolation process in the field of traditional Chinese medicine was established, and the partial differential equation model established could not only reflect the change of percolation extract content, but also predicted the change under different parameters. In view of the difficulty of measuring parameters in the mechanism model, this work established a calculation and screening method for multiple parameters of the percolation process. A small database of axial diffusion coefficient and external mass transfer coefficient was established through literature search, and the optimal parameter combination was determined through parameter combination. This method not only reduced the cost of experiments, but also ensured the accuracy of the model, which can be effectively generalized to other varieties. Aiming at the problems of uneven batches of medicinal materials and easy changes in operating parameters, this work used the established model to establish the design space with material parameters and operating parameters. In the validation experiment, the established model was used for quantitative extraction and endpoint prediction, and the prediction effect was accurate, which proved that the model established in this work had the potential to be applied in the actual production process. The percolation model proposed by this work considers the equipment parameters, process parameters and raw material properties, which will be conducive to the design and amplification of the percolation equipment. This model deepens the understanding of the percolation process of traditional Chinese medicine and provides a new method for the production control of the percolation process.

## Supplementary Information

Supplementary Information.

## Data Availability

All data generated or analysed during this study are included in this published article and its supplementary information files.

## List of symbols

*a*: Specific surface area of medicinal particles
*C*: Concentration of ingredients in solution
${C}_{eq}$: Equilibrium concentrations of components in solution
*C*_w_: Concentration of components in extract
*C*_s_: Concentration of components in solid
${C}_{w}^{0}$: Predicted concentration at the initial time of percolation
${C}_{w}^{*}$: Concentration of ingredients on the outer surface of medicinal material particles
*D*_eff_: Apparent diffusion coefficient
*D*_is_: Volume partition coefficient
*D*_ax_: Axial diffusivity
*d*: Diameter of percolation column
*D*_m_: Molecular diffusion coefficient
*H*: Total bed height of the percolation column
*h*: Bed height from the inlet of the percolation column
*k*: Overall extraction rate constant
*k*_*1*_: Peleg’s rate constant
*k*_*2*_: Peleg’s capacity constant
*K*_x_: Mass transfer coefficient
*k*_*int*_: Internal mass transfer coefficient
*k*_*ext*_: External mass transfer coefficient
*m*: Medicinal mass
*M*_0_: Content of target components in unit mass of medicinal materials
*M*_*B*_: Molar mass of solute
*n*_*i*_: The number of a group in the structural formula
*Pe*: Pecle number
*r*: Medicinal particle radius
*R*^2^: Coefficient of determination
*RE*: Relative error of forecast
RSD: Relative standard deviation
*Sh*: Sherwood number
*Sc*: Schmidt number
*t*: Time
*T*: Experimental temperature
*u*_0_: Flow rate
*V*: Volume
*V*_*A*_: Molar molecular volume of solute at normal boiling point
*V*_*C*_: Critical volume of solute molecules
*V*_*i*_: Group contribution value of a group
*y*_*i*_: The measured value of the i point in the percolation curve
$\overline{{y }_{i}}$: The average value of the measured values of all points on the percolation curve
$\widehat{{y}_{i}}$: The predicted value of the i point in the percolation curve
*α*: Expansion coefficient of medicinal materials
*ρ*: Solution density
*μ*: Solution viscosity coefficient
*Ф*: Association parameters of solvents
*ε*: Bed porosity
