# Supplementary material for: Mechanism modeling and application of Salvia miltiorrhiza percolation process
Source: Sci Rep. 2023 May 23;13:8311. doi: 10.1038/s41598-023-35529-2 (PMC10205710; doi:10.1038/s41598-023-35529-2)
Supplement: Supplementary file 1 — Supplementary Information. [file 41598_2023_35529_MOESM1_ESM.docx]

**Supplementary material**

D_m_ can be calculated by Formula(S1).

| $D_{m}=7.4\times{10}^{-8}\frac{\left( M_{B}\emptyset\right)^{0.5}T}{\mu V_{A}}$ | (S1) |
| --- | --- |

Where, *V_A_* represents molar molecular volume of solute at normal boiling point, which can be calculated by Formula (S2). *M_B_* represents molar mass of solute, *Ф* represents association parameters of solvents (Water is 2.6), *T r*epresents experimental temperature. In this paper, the room temperature was 298.15 K.

| $V_{A}=0.285V_{c}^{1.048}$ | (S2) |
| --- | --- |

Where, *V_C_* represents Critical volume of solute molecules, which can be calculated by group contribution method ^[^[^29^](#_ENREF_29)^]^. The calculation formula is shown in Formula (S3).

| $V_{c}=3.93949+10.90389\sum n_{i}\Delta V_{i}+\frac{3.66576}{\sum n_{i}\Delta V_{i}}-\frac{6.99633}{\left( \sum n_{i}\Delta V_{i} \right)^{2}}\left( {{cm}^{3}}/mol \right)$ | (S3) |
| --- | --- |

Where, *n_i_* represents the number of a group in the structural formula. *V_i_* represents group contribution value of a group. The contribution value of each group can be found in the literature^[^[^29^](#_ENREF_29)^]^.
